# Editorial: Beyond Eating and Body Image Disturbances: Cultural, Transcultural and Accultural Perspectives

**DOI:** 10.3389/fpsyg.2019.02601

**Published:** 2019-11-15

**Authors:** Antonio Cepeda-Benito, Silvia Moreno-Domínguez

**Affiliations:** ^1^Department of Psychological Science, University of Vermont, Burlington, VT, United States; ^2^Department of Psychology, Universidad de Jaén, Jaén, Spain

**Keywords:** body image, body dissatisfaction, eating disorders, transcultural, acculturation, IRAP, palatable foods, food cravings

Body dissatisfaction (BD), a constellation of painful thoughts, feelings, and overall negative evaluation a person has about their own weight, body shape, and/or physical appearance, is a high-risk factor for the emergence and maintenance of eating disorders (ED). Greater understanding about the etiology of BD and the mechanisms that tie BD and ED is needed to improve prevention and treatment outcomes.

Interest in eating-disorders research began in the 1960s, grew rapidly through the late 1980s, and continued to snowball though the 1990s and the first decade of the twenty-first century (Valderrama-Zurián et al., 2017). This research has shaped the idea that BD and ED are the result of Western (Anglo) cultural values present in economically developed countries; and present also in underdeveloped countries among those of high socioeconomic status (SES; Miller and Pumariega, 2001; Swami et al., 2010). Not surprisingly, BD and ED research has historically been carried out almost exclusively with Anglophone populations. BD and ED research with non-Anglos and people of color began to emerge in the 1990s and progressively grew throughout the beginning of the twenty-first century, but this trend reversed its ascending trajectory and has been declining for over 10 years (Cepeda-Benito, unpublished data). To compensate for this downward, problematic trend, we present seven studies that examine BD and EDs comparatively across or within culturally diverse, non-Anglo samples.

The nationalities or residency of the participants represented in this Research Topic spans six different countries: Argentine (Moreno-Domínguez et al.), Brazil (Chapuis-de-Andrade et al.), China (Chen et al.), Germany (Wilhelm et al.), Spain (Hernández-López et al.; Moreno-Domínguez et al.; Ramos et al.) and the USA (Akoury et al.). Two of these studies recruited minorities within economically developed countries: Muslims in Germany (Wilhelm et al.), and Asian Americans in USA (Akoury et al.).

Two studies used path analytic tools to test the hypothesis that cultural variables play a unique role to predict BD and ED symptoms. Akoury et al. and Moreno-Domínguez et al. replicated the finding that internalization of the thin-ideal partially mediates the relationship between thin awareness and BD (Warren et al., 2005). Akoury et al. also found that heightened acculturative stress predicted disordered eating and fully mediated a significant, inverse relationship between biculturalism and ED symptoms. These findings suggest that the degree to which an individual identifies with both their minority origins and the dominant culture where they live protects against ED symptoms by reducing acculturative stress (the challenge of living in an extraneous culture).

Moreno-Domínguez et al. found that both body mass index (BMI) and nationality (Argentine vs. Spanish) moderated the positive relationship between internalization of the thin ideal and BD. Thin-deal internalization predicted BD in both samples, but the strength of the relationship between internalization and BD was stronger among Spaniards than Argentines. The strength of the relationship between internalization and BD also increased with BMI in both samples. Their findings suggest that distance from Western modernization protects against BD, but not against perceived pressure to be thin or internalization of the thin-ideal (as both samples scored similarly in both measures).

Wilhelm et al. compared Muslim, Christian and atheist women across BD-related measures, and also compared their self-reported reactivity to media images of thin women vs. neutral images. Compared to Christian and atheist women, veiled Muslim women reported higher positive body image scores, lower pressure to be thin, lower internalization of the thin ideal, and lower tendencies to engage in appearance social-comparisons. However, reactivity to images of thin women vs. neutral objects did not differ across groups. Their findings suggest that veiled Muslim women may be at lower risk for BD because they experience lower pressure to be thin and encounter less opportunity to engage in upward social comparisons (see also Moreno-Domínguez et al., 2019).

Hernández-López et al. note that whereas self-report research consistently shows robust pro-thin/anti-fat attitudes in the general population, this phenomenon is not consistently observed when investigators measure implicit attitudes (attitudes manifested automatically and therefore not easily manipulated by self-presentation biases). Using the Implicit Relational Assessment Procedure (IRAP) they found that implicit attitudes toward others' body size are moderated by the individual's own level of BD. Whereas, BD women with low BD had equally positive implicit attitudes toward thin and overweight women, women with high BD showed an implicit preference for female thinness and a neutral attitude toward overweight women.

Individuals who diet may experience conflict between their desire to savor tasty foods and their desire to attain thinness. The study by Chen et al. represents a novel and successful attempt to index the cognitive load of ambivalence between appetitive-enjoyment vs. body-shape goals. In a Stroop-like task, Chinese women who self-identified as restrictive dieters experienced greater cognitive interference when classifying “thin-words” presented alongside food pictures than “food-words” presented with images of thin female bodies. Their novel results suggest that dieters may have a stronger attentional bias or pull toward images of palatable foods than images of thin women, which could in turn explain a hypothetical link between dieting, food cravings and binge eating (c.f., Moreno et al., 2009).

The remaining two studies analyzed nationally representative data sets. Chapuis-de-Andrade et al. analyzed data from a sample of 27,501 Brazilian participants and reported the odds ratios for six different compensatory behaviors as a function of race, educational attainment and religion. Ramos et al. analyzed a data set of 4,531 Spanish adolescent and found that BD was a robust predictor of anxiety, depression, social withdrawal, and somatic complains. Congruent with previous research, affluency, or high SES increased the risk of BD.

The current Research Topic generated a diverse collection of studies on BD and ED. The collection included participant representation from three continents and six countries, and the studies were also theoretically and methodologically diverse. Three studies used path-analytic technics to test mediational and moderating links to BD and ED; two studies operationalized constructs related to BD and ED using objective cognitive-load measures (methodology that provides an important perspective with which self-report data can be contrasted); and the two nationally representative studies provided important comparative benchmarks for past and future studies. We are optimistic culturally diverse research will continue to produce important contributions to the field.

## Author Contributions

SM-D provided an initial summary of the studies. AC-B drafted the manuscript.

### Conflict of Interest

The authors declare that the research was conducted in the absence of any commercial or financial relationships that could be construed as a potential conflict of interest.
